# Explaining the Insights Obtained from the BRANDO Database

**DOI:** 10.1055/s-0045-1814438

**Published:** 2026-01-25

**Authors:** Joseph R. Berger, Thomas Pisano

**Affiliations:** 1 University of Pennsylvania, Perelman School of Medicine, Department of Neurology, Philadelphia, Pennsylvania, United States

In this retrospective, cross-sectional study of epidemiological data collected in Brazil for the Collaborative Latin American Database for Multiple Sclerosis (BRANDO), the authors examine differences in a variety of parameters across four regions of Brazil. 1 These four regions were the Southeast, Northeast, South and Midwest. As the first nationwide MS epidemiology and healthcare access study from Brazil, leveraging a multicenter registry represents an important step forward for understanding the diversity of MS across the country and generating real-world data that has long been available in higher-income nations. 2

**Figure 1:**
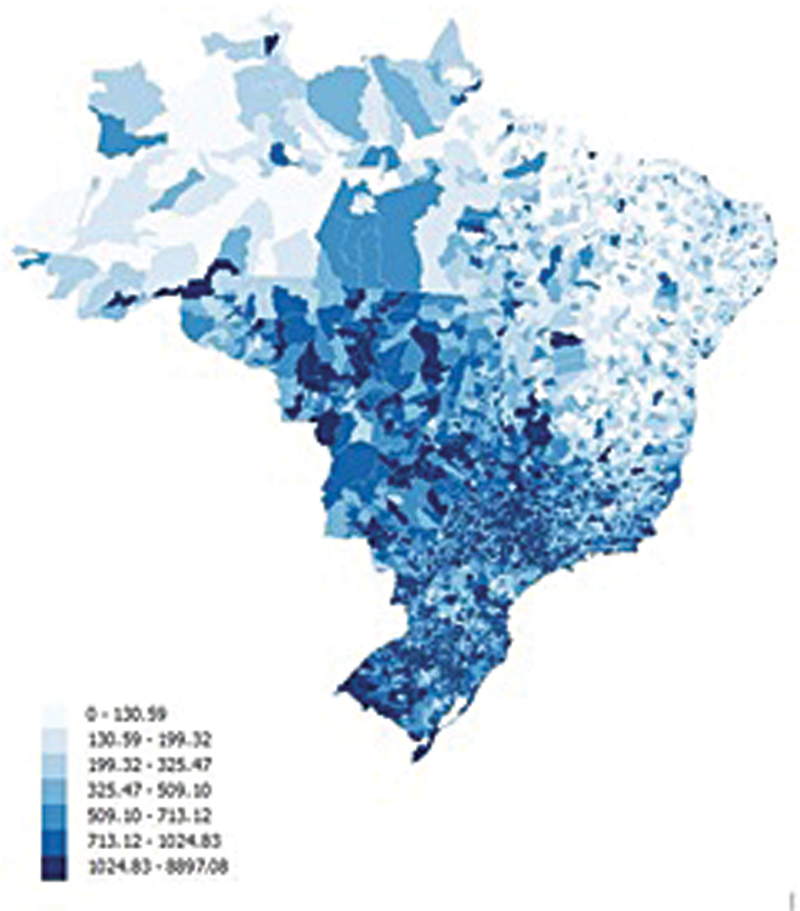
Average income of taxpayers reported from 5570 municipalities in Brazil. 4

With rare exception, such as the gender differences observed between regions, their findings are unsurprising and likely reflect broader socioeconomic patterns in wealth distribution. Brazil is a country with great income disparity; the richest 1% of the population has 13% of the household income. 3 The map below shows the average income of taxpayers by region ( Figure 1 ). 4 Persons living in the Southeast and Midwest region have the highest income. Persons living in these higher income areas are more likely to be better educated and have greater access to medical care. 5 In addition to income, regional differences in neurologist density (and MRI availability) also strongly influence diagnostic timeliness and treatment access. 6

Among the findings from this study was a higher prevalence of relapsing-remitting multiple sclerosis in the Southeast and Midwest regions. In a population with greater access to medical care as well as one that is better educated, one would anticipate a greater proportion of patients being diagnosed earlier and, therefore, being more likely to have relapsing-remitting disease rather than progressive disease, generally associated with more advanced illness. The investigators also found that the shortest time to diagnosis from disease onset to first access of disease-modifying therapy occurred in the Midwest region of the country. This, too, may simply correlate with the relative wealth in that region. These interpretations, while plausible, should be recognized as hypotheses because the study did not directly measure socioeconomic variables or healthcare infrastructure.

The obverse is demonstrated in the Northeast region of the country which had the highest rates of progressive disease. The authors noted that 63.7% of the MS population in the Northeast region had an Expanded Disability Status Scores (EDSS) of 4.0 or higher. One could surmise that this was the consequence of a delay in diagnosis and in the early initiation of effective treatment. The Northeast also had higher proportions of mixed-race and African-descendant individuals, which mirrors census patterns and may warrant future investigation given global evidence that some ethnic groups accumulate disability more rapidly. 7

Multiple studies in the United States have demonstrated that lower education and income are strongly associated with reduced access to medical care. 8 9 10 This occurs as a consequence of financial barriers, lack of insurance and systemic inequities. Surprisingly, in a study that looked at medical care access in 28 countries (not including Brazil), no statistical correlation between access and public health care expenditure could be demonstrated. 11 This finding may be explained by high relative out-of-pocket costs, the overall financial strain, and negative medical care experiences of persons with lower income. 12

In treating multiple sclerosis, early diagnosis and prompt initiation of a highly effective disease-modifying therapy have been demonstrated to decrease relapse rates, disease related disability, and the cost of multiple sclerosis. A study from the North American Research Committee demonstrated that for every successive 5-year period between 1980 and 2004, the time from symptom onset to diagnosis of multiple sclerosis substantially declined. 13 Undoubtedly, following broad adoption of the McDonald 2024 criteria, 14 the diagnosis will be rendered with even greater alacrity. Numerous studies have demonstrated the benefit of early treatment initiation on disease progression 14 15 16 17 18 and on the ultimate cost of the illness. 19 Retrospective studies have also demonstrated the value of initiating high dose therapy as opposed to an escalatory strategy. 20 21

While this study provides valuable national insight, it also highlights limitations that are important for interpreting the findings. Data availability varied substantially across outcomes, the North region of the country was not represented, and diagnoses across centers likely relied on a mix of McDonald 2010 and 2017 criteria rather than a single uniform standard. These factors may partly explain regional differences in diagnostic delay, relapse topography, and phenotype assignment. Additionally, the retrospective nature of registry entry may introduce bias toward MRI-confirmed events and limit the precision of topography estimates, as the authors themselves note. Their discussion also cites work by Nathoo and colleagues on MRI differences across racial and ethnic groups, underscoring the need for more diverse imaging data. 22

The goal for physicians in Brazil, as elsewhere, is to optimize the care of multiple sclerosis. Facilitating access to medical care, establishing the diagnosis as soon as possible, and promptly initiating high efficacy therapy once the diagnosis has been made remain core principles. Beyond these clinical steps, the findings of this study highlight opportunities for system-level improvements: expanding and standardizing the BRANDO registry to ensure representation of all regions, incorporating socioeconomic and health-system variables, and increasing MS-specific education among primary care providers to improve early referral and symptom recognition. 23 These measures will be important in addressing the disparities that exist within the country.
